# Supplementary material for: Global coastal exposure patterns by coastal type from 1950 to 2050
Source: Camb Prism Coast Futur. 2025 Jun 16;3:e12. doi: 10.1017/cft.2025.10001 (PMC12337604; doi:10.1017/cft.2025.10001)
Supplement: Nyberg et al. supplementary material [file S2754720525100012sup001.docx]

**Supplementary Material:**

**Global Coastal Exposure Patterns by Coastal Type from 1950 to 2050**

Bjorn Nyberg^1,2,3^, Albina Gilmullina^1^, William Helland-Hansen^1,2^, Jaap Nienhuis^4^, Joep Storms^5^

^1^ Department of Earth Sciences, University of Bergen

^2^ Bjerknes Centre for Climate Research, Bergen Norway

^3^ Currently - 7Analytics, Innovation Centre, Bergen Norway

^4^ Department of Physical Geography, Utrecht University

5 Dept Geoscience and Engineering, Delft University of Technology, Delft, the Netherlands

**Supplementary Figures and Tables**


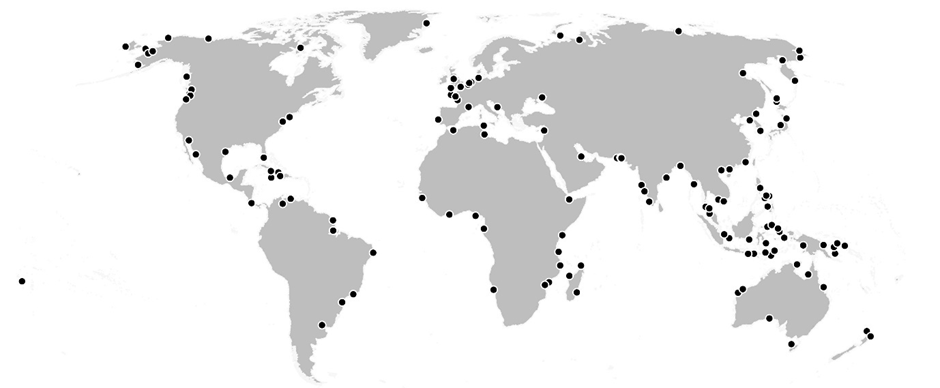


**Supplementary Figure S1** – Location of 140 randomly selected regions, 20 for each coastal type, used for the confusion matrix agreement correlation in Extended Figure E3.


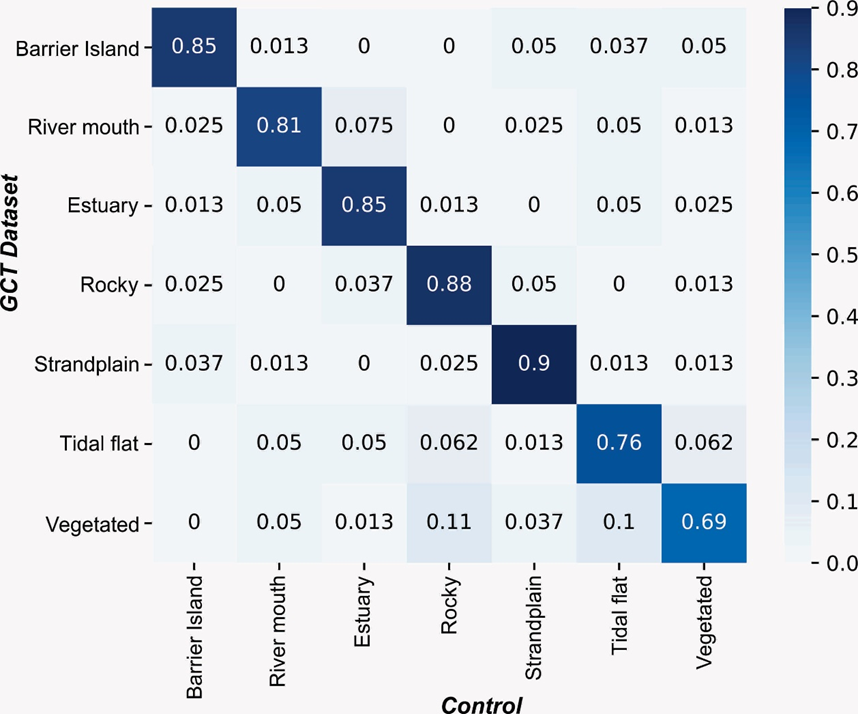


**Supplementary Figure S2** – Confusion matrix showing the correlation between the GCT Dataset and 4 independent geomorphology experts’ interpretation of the coastal type for a total of 560 locations (140 x 4; see Extended Figure E2).

**
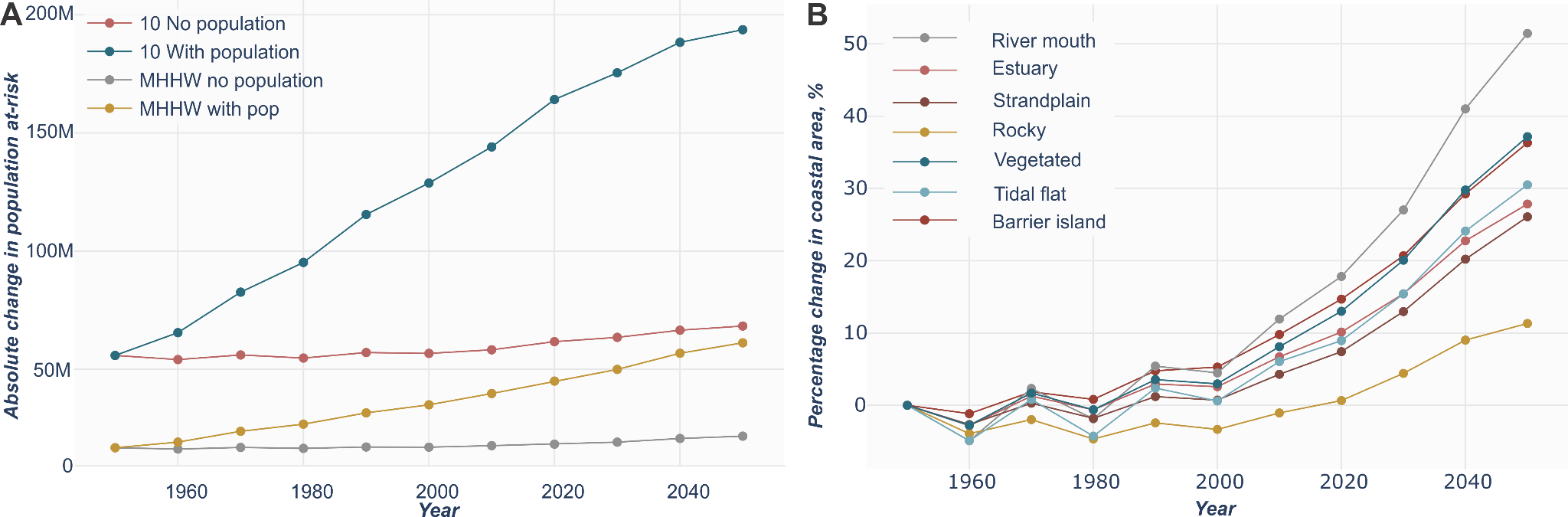
**

**Supplementary Figure S3** - A) Absolute change in population at risk for two scenarios of with and without population change since 1950 for a MHHW and 10-year storm surge level. B) Percentage change in coastal area inundated from a MHHW level by coastal type.


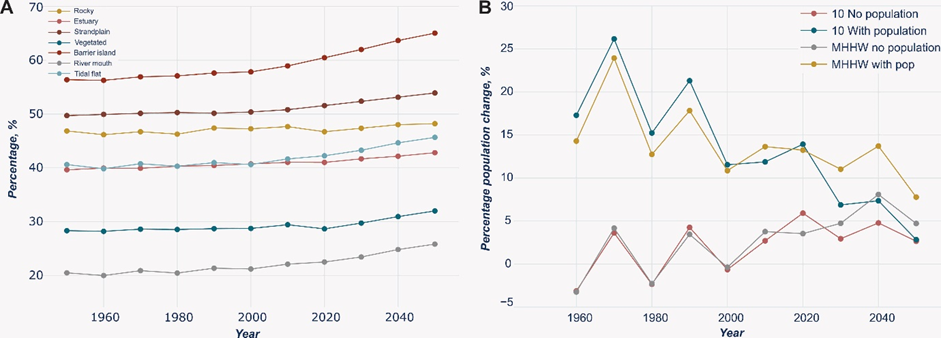


**Supplementary Figure S4** – A) Shows the percentage of the inundated area for a 1 in 10-year storm surge flood that is attributed to MHHW level by coastal type from 1950 to 2050. B) Shows the annual percentage change in population at risk based on four scenarios from 1950 to 2050 for a MHHW and 10-year storm surge event.

**Supplementary Table S1:** Table showing the geospatial dataset, source and purpose in the classification of the GCT dataset.

| **Dataset** | **Source** | **Purpose** |
| --- | --- | --- |
| Global River Dataset | *Caldwell et al., 2022* | Define river outputs |
| DINAS Coastline | Vafeidis et al., 2008 | Coastline polyline |
| Beach Distribution | Mao et al., 2022 | Distribution of beach, wetland vs rocky |
| WCMC008_CoralReef 2018 | UNEP-WCMC, WorldFish Centre, WRI, TNC (2021). | Global coral reef distribution |
| Bing Aerial | Microsoft Bing © | Satellite imagery |
| Worldwide Typology of Nearshore Coastal Systems | Dürr et al., 2011 | Distribution of lagoons |

**References**

[**Caldwell RL, Edmonds DA, Baumgardner S, Paola C, Roy S and Nienhuis JH** (2019) A global delta dataset and the environmental variables that predict delta formation on marine coastlines. *Earth Surface Dynamics* **7**(3), 773–787. https://doi.org/10.5194/esurf-7-773-2019.](https://www.zotero.org/google-docs/?23o2ea)

[**Dürr H, Laruelle G, Kempen C, Slomp C, Meybeck M and Middelkoop H** (2011) Worldwide Typology of Nearshore Coastal Systems: Defining the Estuarine Filter of River Inputs to the Oceans. *Estuaries and Coasts* **34**(3), 441–458. https://doi.org/10.1007/s12237-011-9381-y.](https://www.zotero.org/google-docs/?23o2ea)

[**Mao Y, Harris DL, Xie Z and Phinn S** (2022) Global coastal geomorphology – integrating earth observation and geospatial data. *Remote Sensing of Environment* **278**, 113082.](https://www.zotero.org/google-docs/?23o2ea) https://doi.org/10.1016/j.rse.2022.113082[.](https://www.zotero.org/google-docs/?23o2ea)

**Microsoft.** 2024**.** *Bing Maps Imagery*. Retrieved from https://www.bing.com/maps

**UNEP-WCMC, WorldFish Centre, WRI, TNC** (2021) Global distribution of coral reefs, compiled from multiple sources including the Millennium Coral Reef Mapping Project. Version 4.1, updated by UNEP-WCMC. Includes contributions from IMaRS-USF and IRD (2005), IMaRS-USF (2005) and Spalding et al. (2001). Cambridge (UK): UN Environment Programme World Conservation Monitoring Centre. Data DOI: https://doi.org/10.34892/t2wk-5t34[.](https://www.zotero.org/google-docs/?23o2ea)

[**Vafeidis AT, Nicholls RJ, McFadden L, Tol RSJ, Hinkel J, Spencer T, Grashoff PS, Boot G and Klein RJT** (2008) A New Global Coastal Database for Impact and Vulnerability Analysis to Sea-Level Rise. *Journal of Coastal Research* 917–924. https://doi.org/10.2112/06-0725.1.](https://www.zotero.org/google-docs/?23o2ea)
